# Evaluation of sarolaner and spinosad oral treatments to eliminate fleas, reduce dermatologic lesions and minimize pruritus in naturally infested dogs in west Central Florida, USA

**DOI:** 10.1186/s13071-017-2328-9

**Published:** 2017-08-17

**Authors:** Michael W. Dryden, Michael S. Canfield, Emily Niedfeldt, Amanda Kinnon, Kimberly Kalosy, Amber Smith, Kaitlin M. Foley, Vicki Smith, Todd S Bress, Nicole Smith, Mike Endrizzi, Joyce Login

**Affiliations:** 1 Department of Diagnostic Medicine/Pathobiology, Kansas State University, Manhattan, KS 66506 USA; 2 Animal Dermatology South, 7741 Congress Str, New Port Richey, FL 34653 USA; 3 Zoetis US LLC, 100 Campus Drive, W4668, Florham Park, NJ 07932 USA; 4 GBBM Consulting, 16036 Eagle River Way, Tampa, FL 33624 USA

**Keywords:** *Ctenocephalides felis felis*, Cat flea, Dogs, Field study, Sarolaner, Flea, Flea control, Spinosad, Flea allergy dermatitis, Flea bite dermatitis, Pruritus, Atopic dermatitis

## Abstract

**Background:**

An in-home investigation of naturally flea infested dogs was conducted in West Central Florida, USA to evaluate and compare the effectiveness of two different oral flea adulticides to control flea infestations, minimize dermatologic lesions and reduce pruritus over an 8-week period.

**Methods:**

Twenty-nine dogs living in 19 homes and another 26 dogs residing in 16 different homes were orally administered either a sarolaner or spinosad chewable, respectively on day 0 and once between days 28–30. Products were administered by study personnel according to label directions. Flea populations on dogs were estimated using visual area counts and flea infestations in the indoor premises were assessed using intermittent-light flea traps on days 0, 7, 14, 21 and once between days 28–30, 40–45, and 56–60. Assessments of dermatologic lesions were conducted monthly during the study and severity of pruritus was evaluated throughout the study on the same schedule as flea counts were conducted. Concurrent treatments for existing skin disease were not allowed.

**Results:**

The administration of sarolaner or spinosad reduced flea populations on dogs by 99.0% and 97.3%, respectively within 7 days. Flea infestations on the sarolaner- and spinosad-treated dogs were reduced by > 99% at every counting period from day 14 post-treatment through the end of the 8-week study. At the end of the study 96.4 and 92.0% of the dogs treated with sarolaner and spinosad, respectively were flea-free. Flea populations in the indoor premises were also markedly reduced the end of the study, with 100 and 99.8% reductions in flea trap counts in the sarolaner and spinosad treatment groups, respectively. FAD lesion scores, atopic dermatitis lesions scores (CADESI-4) and pruritus severity scores were also markedly improved with both formulations.

**Conclusions:**

An in-home clinical field study conducted during the summer of 2016 in subtropical Florida demonstrated that two-monthly administrations of either sarolaner or spinosad chewables almost completely eliminated flea infestations on dogs and in private residences, while markedly reducing dermatology lesions and pruritus.

## Background

*Ctenocephalides felis felis*, the cat flea, is the most prevalent flea species found worldwide on cats and dogs [1–3]. Hypersensitivity to flea saliva, from flea feeding, often results in flea allergy dermatitis with accompanying pruritus, hair loss, and primary and secondary skin lesions [4]. Additionally, though the volume of the flea blood meal is small, approximately 13.6 μl/day/female flea, severe flea infestations may result in anemia [4, 5]. The cat flea also serves as a vector for several zoonotic diseases as well as serving as the primary intermediate host of *Dipylidium caninum*, a common tapeworm of both dogs and cats [4]. Despite the availability of many effective flea control products, fleas remain an extremely important external parasite of dogs and cats and in-home infestations continue to be a major frustration for pet owners [6, 7].

The isoxazolines are a new class of ectoparasiticides with potent insecticide and acaracide activity. After oral administration, these compounds have a high bioavailability with a rapid elimination of existing flea and tick infestations and protective efficacy for the duration of the dosing interval [7]. Sarolaner (Simparica®), is a novel oral isoxazoline available in a palatable chewable formulation dosed at monthly intervals. Sarolaner is effective in eliminating existing flea infestations on dogs and provides protective control against reinfestation for 35 days [8, 9]. It has been demonstrated that fleas begin to die within 3 to 4 h after sarolaner administration with > 98% elimination of fleas by 8 h and residual efficacy >95.7% within 12 h after reinfestation 35 days post-treatment, which was also sufficient to prevent flea egg deposition during the entire 5-week post-treatment period [10]. Additionally, studies have shown sarolaner to be efficacious in the treatment of *Sarcoptes* and *Demodex* mange mites, as well for the treatment of *Otodectes* ear mites [11, 12].

Goals of an optimal flea control program include rapid elimination of existing flea infestations, rapid cessation of viable flea egg production, and protection against reinfestation during the dosing interval that eventually results in both premise and on-animal flea infestations essentially controlled or eliminated [13]. Several field studies with a wide variety of insecticidal compounds have confirmed these findings [14–27].

The present study was conducted to evaluate the comparative efficacy of sarolaner (Simparica®; Zoetis) chewables and spinosad (Comfortis®; Elanco) oral chewable tablets in eliminating flea infestations on pets and in homes, as well as measuring the reduction of pruritus and skin lesions associated with flea infestations over an eight-week study period in dogs with naturally acquired flea infestations. Spinosad, a spinosyn class insecticide, was chosen as a positive reference control due to its established performance against fleas in previous field studies [21, 25].

## Methods

### Home and pet study inclusion criteria

Thirty-eight homes were selected for inclusion in the study using client referrals from Sunshine Animal Hospital, Tampa, Florida, Animal Dermatology South, New Port Richey, Florida, and advertisements on CRAIGSLIST®

Criteria for home and pet inclusion included: (i) ≥ five fleas on at least one dog at the residence; (ii) ≥ five fleas recovered during a 16–24 h collection period in two light flea traps; (iii) one to five healthy dogs living in the home; (iv) dogs must spend ≥ 12 h/day inside the house; (v) homeowner’s ability to start the study between May 18 and June 4th 2016 and agreeing to remain in the study for 8 weeks; (vi) homeowners agreeing that no other flea control product can be used during the course of the study; (vii) owners agreeing that no other potential flea host (dog, cat, rabbit, ferret, etc.) can be brought into the residence during the study; (viii) dogs and cats cannot be pregnant or nursing; (ix) dogs must be > 6 months of age and > 2.0 kg., and cats need to be at least 8 weeks of age and 1.28 kg; (x) owners willing to complete a questionnaire concerning pet habits, flea control history and personal observations concerning potential flea hosts around their residence; and (xi) no residual topical or oral flea product administered in the previous 30 days (or 7 months if using a Seresto® collar).

### Treatment groups

Pets and homes meeting inclusion criteria were randomly allotted to 1 of 2 treatment groups on Day 0. Household entry numbers (1–38) were assigned a random number by Excel (Excel 2013) and blocked into groups of 2. The highest random number within each block was assigned to Group 1 and lowest to Group 2.

In treatment Group 1 dogs were orally administered a sarolaner (Simparica®; Zoetis) chewable at a minimum dosage of 2 mg/kg. While this study was focused on dogs, cats in these households were also treated topically with selamectin (Revolution®; Zoetis LLC) at a minimal dose of 6 mg/kg.

In treatment Group 2 dogs were orally administered, following a meal, a spinosad (Comfortis®; Elanco) chewable tablet at a minimum dosage of 30 mg/kg. The cats in these households were treated topically with an 11.2% spinetoram spot-on (Cheristin®; 0.77 ml; Elanco) at a minimal dose of 12 mg/kg.

All treatments were administered per label directions using product dose banding on study days -1 or 0, and once between days 28–30. Prior to treatment all animals were weighed and all treatments were conducted by study personnel who were not blinded to treatment groups. All dogs and cats living at a residence were administered group appropriate treatment and no other pet or premises flea treatments were used during the study. Additionally, no antibiotics, corticosteroids, antihistamines, or medicated shampoos were used to alleviate pruritus or skin lesions. There were no restrictions on the animals regarding exposure to rain, swimming, bathing, or movement outdoors.

This study was conducted without a placebo control group. Based on the experience of these authors it is their opinion that the large flea infestations frequently observed on dogs and cats in subtropical Florida preclude the use of a non-treated group. Withholding flea adulticide treatment would be detrimental to the health and welfare of the pets and potentially even humans in these households.

### Flea population assessment

Fleas present in the indoor premises of these homes were assessed using intermittent light traps [28, 29]. A trap was placed in each of two rooms during 16 to 24-h collection periods. Selection of the two rooms was based on where the dog(s) spent the majority of their time or where owners had observed fleas. Throughout the study traps were placed in the same location within those rooms at every counting period. Species, sex and number of fleas collected on the sticky-sheets of the traps were recorded.

The number of fleas on each pet was estimated using a previously described and validated area count methodology [30]. Fleas were counted in five areas on each animal; dorsal midline, tail head, left lateral, right lateral, and inguinal region. Counts in each of these five areas were limited to one minute and conducted by parting the hair against the lay using both hands until the area was covered. Flea numbers in each of these areas was capped at 50; therefore, the maximum total area flea count was 250. All on-animal and premises flea counts were conducted ±1 day on days 0, 7, 14, 21, then once between days 28–30, 40–45, and 56–60. Personnel conducting pet and premises flea counts were not blinded to treatment groups.

### Evaluation of skin disease and pruritus

The severity of pruritus of the qualifying dog(s) in each home was evaluated by a pet owner on days 0, 7, 14, 28-30, 40–45 and 56–60 using a previously validated non-numeric scale [31, 32]. One owner in each home was provided a form with 6 written descriptions of increasing pruritus severity, from “Normal dog - I do not think itching is a problem” through “Extremely severe itching.” [31, 32]. Owners were asked to place a single line on the form corresponding to the severity of pruritus of their dog(s). Following the pruritus rating by the owner, study personnel placed a numeric scale running from 0 to 10 on the form and a number corresponding to the owner’s subjective assessment of the pruritus level was recorded. Owners were not informed of the numerical score applied to their dog’s level of pruritus during the study. The same owner in each household assessed the pruritus level of the dog(s) throughout the study.

Clinical dermatologic observations were made on days 0, 30 and 60 of the study (± 3 days) of all qualifying dogs in the homes by a board-certified veterinary dermatologist (MC) who was blinded to treatment groups and all flea counts. Two different assessment tools were used to evaluate skin lesions during the study. Skin lesions potentially associated with flea allergy dermatitis (FAD) were evaluated using a flea bite hypersensitivity severity scale that assesses crusts, papules, excoriation, scale, erythema and alopecia [33]. Severity of each of these dermatologic categories was scored using a scale from 0 to 3: 0, no signs; 1, mild; 2, moderate; 3, severe. There were three areas on the dog’s body that were evaluated: (i) dorsum, from the withers to the base of the tail; (ii) left and right lateral thorax, just caudal to the elbow and extending to the last rib; and (iii) “flea triangle”, including the dorsal lumbosacral region, caudomedial thighs, and ventral abdomen [33]. Scores for each of the six dermatologic categories at each of the three body sites were totaled to provide the dog’s overall FAD assessment score. The second dermatologic lesion assessment tool used by the boarded-dermatologist was the canine atopic dermatitis extent and severity index scoring system (CADESI-4) [34]. While CADESI-4 has been used primarily as a means to assess the skin in regions of dogs more commonly associated with atopic dermatitis patients, there is overlap in the two scoring systems and we wanted to compare the two scoring systems simultaneously.

### Data analysis

This study was conducted as a randomized complete block design. Statistical analyses were performed on flea counts in traps, flea counts on animals, CADESI scores, total dermatology report scores and pruritus scores.

Day 0 data for each variable were analyzed by a linear mixed model approach in order to make sure that both treatment groups were equal at the start of the study. If there was a significant difference between treatment groups on Day 0 for any given variable, Day 0 data would have been used as a covariate in the analysis. All variables were then analyzed by a linear mixed model approach for repeated measures. Flea counts were transformed by the log_10_(count ±1) transformation prior to any statistical analysis. Geometric means and % reduction, based on geometric means for flea counts in traps and on pets were calculated separately as follows:

Geometric mean = (10^Log-transformed count^) – 1 and % Reduction = (Day 0 Geometric Mean – Day × Geometric Mean)/(Day 0 Geo. Mean) × 100, where x = Day 7, 14, 21, 28, 40 or 54.

Using the SAS Proc Mixed Procedure (SAS 9.3, Cary NC) Day 0 data were analyzed with a model that considered the fixed effects of treatment and the random effects of block and the residual error. No significant differences between groups were observed on any Day 0 variables. Therefore, no covariate was needed in the repeated measures analysis.

The repeated measures analysis for all variables used a model that considered the fixed effects of treatment, day and the interaction of treatment-by-day and the random effects of block and the residual error. Time was the repeated factor. The covariance structure in the repeated measures analysis was investigated using five structural assumptions, namely, compound symmetry, spatial power SP, first order autoregressive, heterogeneous first order autoregressive and unstructured. The assumption giving the minimum value of the Akaike’s Information Criterion was selected in the final analysis. Treatment Least Square Means (LSMeans) were calculated for each group. Comparison of LSMeans were performed by the two-sided Student’s *t*-test at the 5% level of significance.

If the treatment-by-day interaction was significant at the 5% level, group comparisons were assessed for each day, using the same model that was used in the analyses for Day 0.

## Results

While 38 households were enrolled in the study, only 35 remained in the study beyond day 14. Three households, all in the spinosad treatment group did not remain in the study beyond day 14; data from those households were not included. In two homes following the initial visit and treatment the owners did not respond to further attempts to set up appointments. In the other home the owner moved out of the study area. Several additional households and/or dogs within a home remained in the study up to the 40–45 day assessment period but were unavailable for the final assessment between days 56–60. Data from those households through days 40–45 were utilized in product assessment. In the sarolaner group, data from one home with one qualifying dog was not collected on the last assessment day because the owner would not return phone calls to make an appointment. In the spinosad group, one dog was lost on the last day of the study because the owner became agitated and uncooperative during the visit.

In the 19 homes in the sarolaner group that remained in the study beyond days 28–30 there were 29 dogs (mean 21.1 kg; range 1.8–47.7 kg) enrolled. On day 0 dogs were administered a mean oral dose of 2.8 mg/kg (range 2.0–3.7 mg/kg) sarolaner. There were also 14 additional dogs in these households that were treated but did meet all qualifying criteria. Typically, these dogs either had fewer than 5 fleas, did not spend the majority of their time inside the home, or could not be safely handled for flea counts or dermatologic assessments. There were therefore, 43 qualifying and non-qualifying dogs treated with sarolaner on day 0. There were only 2 cats that qualified for inclusion in the study in these homes and an additional 12 cats that did not qualify for reasons similar to the non-qualifying dogs. Given the low number of qualifying cats, only dog flea count data were used in this study.

Sixteen (16) homes in the spinosad treatment group remained in the study for at least 6 weeks. These homes contained 26 (mean 11.4 kg; range 2.0–55.7 kg) qualifying dogs. On day 0 the dogs were administered a mean oral dose of 43.8 mg/kg (range 30.2–59.2 mg/kg) spinosad. There were 6 other non-qualifying dogs in these homes. There were a total of 32 qualifying and non-qualifying dogs residing in the 16 homes that were treated with spinosad on day 0. There was only 1 cat in these residences that qualified for inclusion in the study and an additional 9 cats that did not qualify for reasons similar to the non-qualifying dogs. As above, only dogs were used in flea count analyses.

On day 0, dogs treated with sarolaner had a geometric mean of 24.1 (range 5–119) fleas in the area counts (Table 1). Dogs administered spinosad had a geometric mean of 25.7 (range 7–128) fleas in area counts on day 0. Seven days following the administration of sarolaner or spinosad flea counts were reduced by 99.0 and 97.7%, respectively (Table 1). By days 56–60 the flea counts in the sarolaner treatment group were markedly reduced by 99.9% (Table 1). Spinosad also showed reduced flea populations, with 99.8% control on day 56–60 (Table 1). Sarolaner and spinosad oral treatments significantly reduced flea counts on dogs across time (*P* < 0.0001). The flea counts of the two treatment groups were not significantly different on any of the post-treatment count days (*P* = 0.1968).

**Table 1:** On-animal flea counts in naturally infested homes when dogs were administered either Sarolaner or Spinosad oral treatments

Treatment group	No. of dogs on day 0		Days post-treatment^a^						
			Day 0	Day 7	Day 14	Day 21	Day 28–30	Day 40-45	Day 56-60
Sarolaner^b^	29	Geomean^d^	24.12	0.24	0.20	0.07	0.07	0.00	0.02
		Range	5–19	0–1	0–2	0–3	0–1	0–0	0–1
		% control^e^		99.00	99.18	99.69	99.69	100	99.90
		%	0	68.97	75.86	93.10	89.66	100	96.43
		(#) dogs with no fleas	(0/29)	(20/29)	(22/29)	(27/29)	(26/29)	(29/29)	(27/28)
Spinosad^c^	26	Geomean	25.71	0.60	0.21	0.19	0.17	0.08	0.06
		Range	7–128	0–6	0–8	0–3	0–6	0–1	0–1
		% control		97.26	99.20	99.25	99.35	99.68	99.78
		%	0.0	61.54	84.62	80.77	84.62	88.46	92.0
		(#) dogs with no fleas	(0/26)	(16/26)	(22/26)	(21/26)	(22/26)	(23/26)	(23/25)

^a^In both groups, dogs were treated on day 0 and once between days 28–30

^b^Dogs were orally administered Sarolaner chewables (Simparica® Zoetis) according to label directions

^c^Dogs were orally administered Spinosad flavored tablets (Comfortis® Elanco) according to label directions

^d^Geometric mean numbers of fleas in visual area counts on pets

^e^{(Day 0 geometric mean animal area flea counts – day x geometric mean animal area flea counts) / day 0 geometric mean animal area flea counts)} × 100

No significant differences between the two groups were observed, for any day, based on the repeated measures analysis (*P* = 0.1969)

The number of flea-free dogs observed following administration of sarolaner and spinosad were similar (Table 1). Following a single oral dose of sarolaner 75.9% (22/29) of dogs had no fleas observable in area counts on day 14 and only a single flea was found on one dog at the final 56–60 day flea count, with 96.4% (27/28) of dogs flea free. Similarly, 84.6% (22/26) of the dogs in the spinosad treatment group had no fleas in area counts on day 14 and one flea was found on each of two dogs at the final 56–60 day flea count, with 92.0% (23/25) of dogs flea free (Table 1).

During the 8-week study at total of 2606 fleas were collected in the intermittent light traps in the 35 residences and all were identified as *Ctenocephalides felis felis*, the cat flea. A geometric mean of 29.4 (range 6–1163) and 21.0 (range 6–138) fleas were collected in traps on day 0 in homes in the sarolaner and spinosad treatment groups, respectively (Table 2). Premises flea trap counts were reduced by 96.1 and 100% on days 28–30 and 56–60, respectively in the homes where dogs were treated with sarolaner (Table 2). Trap flea counts were similarly reduced in the homes where dogs were administered spinosad, with reductions of 94.9 and 99.8% occurring on days 28–30 and 56–60, respectively (Table 2).

**Table 2:** Fleas recovered in premises flea traps in naturally infested homes when dogs were administered either Sarolaner or Spinosad oral treatments

Treatment group	No. of homes on day 0		Days post-treatment^a^						
			Day 0	Day 7	Day 14	Day 21	Day 28–30	Day 40–45	Day 56–60
Sarolaner^b^	19	Geomean^d^	29.39	4.08	4.14	1.25	1.14	0.26	0.00
		% control^e^		86.11	85.92	95.74	96.12	99.13	100
		Range	6–1163	0–164	0–61	0–58	0–39	0–2	0–0
Spinosad^c^	16	Geomean	20.99	4.65	2.38	1.27	1.07	0.04	0.04
		% control		77.83	88.65	93.95	94.91	99.80	99.79
		Range	6–138	0–49	0–16	0–16	0–4	0–1	0–1

^a^In both groups, dogs were treated on day 0 and once between days 28–30

^b^Dogs were orally administered Sarolaner chewables (Simparica® Zoetis) according to label directions

^c^Dogs were orally administered Spinosad flavored tablets (Comfortis® Elanco) according to label directions

^d^Geometric mean numbers of fleas recovered in two intermittent light flea traps averaged within households

^e^{(Day 0 geometric mean trap flea counts – day x geometric mean trap flea counts) / day 0 geometric mean trap flea counts)} × 100

Dogs administered sarolaner and spinosad had mean flea bite hypersensitivity severity lesion scores of 11.0 (range 1–30) and 13.6 (range 3–32), respectively on day 0 (Table 3). Following two monthly treatments the flea bite hypersensitivity severity lesion scores were reduced to 3.7 (66.2% improvement) and 5.2 (62.1% improvement), in the sarolaner and spinosad treatment groups respectively by days 56 to 60 (Table 3). Statistically significant reductions in flea bite hypersensitivity lesion scores were observed in both treatment groups (*P* < 0.0001), but there was no statistically significant difference between treatment groups (*P* = 0.2086).

**Table 3:** Assessment of skin lesions using a flea bite hypersensitivity severity scale for dogs naturally infested with fleas and administered either Sarolaner or Spinosad oral treatments

Treatment group		Days post-treatment^a^		
		Day 0	Day 30	Day 60
Sarolaner^b^	# Dogs	29	29	28
	Mean FAD Score^e^	11.00	5.38	3.71
	SD	8.19	4.97	4.03
	Range	1–30	0–21	0–15
	Reduction (%)^e^		51.10	66.23
Spinosad^c^	# Dogs	26	26	25
	Mean FAD Score^d^	13.62	6.73	5.16
	SD	7.42	6.51	5.17
	Range	3–32	0–26	0–24
	Reduction (%)		50.56	62.10

*Abbreviation: SD* standard deviation

^a^In both groups, dogs were treated on day 0 and once between days 28 – 30

^b^Dogs were orally administered Sarolaner chewables (Simparica® Zoetis) according to label directions

^c^Dogs were orally administered Spinosad flavored tablets (Comfortis® Elanco) according to label directions

^d^Arithmetic mean Flea Allergy Dermatitis lesion score [33]

^e^{(Day 0 arithmetic mean FAD score – day x arithmetic mean FAD score)/day 0 arithmetic mean FAD score)} × 100

No significant differences between the two groups were observed, for any day, based on the repeated measures analysis (*P* = 0.2086)

Similar reductions in CADESI-4 lesion scores were also observed. On study entry day 0, dogs administered sarolaner or spinosad had mean total CADESI-4 lesion scores of 35.4 (range 4–94) and 32.7 (range 1–91), respectively (Table 4). On the final assessment conducted between days 56 to 60, CADESI-4 lesion scores had improved to 11.5 (67.4% improvement) in dogs administered sarolaner and 12.3 (62.4% improvement), in dogs administered spinosad (Table 4). Regardless of treatment administered, statistically significant reductions in CADESI-4 scores (*P* < 0.0001) occurred over time, but no statistically significant difference between treatment groups (*P* = 0.8236) was observed.

**Table 4:** Assessment of skin lesions using the Canine Atopic Dermatitis Extent and Severity Index-4 (CADESI-4) scale for dogs naturally infested with fleas and administered either Sarolaner or Spinosad oral treatments

Treatment group		Days post-treatment^a^		
		Day 0	Day 28–30	Day 54-60
Sarolaner^b^	# Dogs	29	29	28
	Mean CADESI - 4 Score^d^	35.41	17.62	11.54
	SD	22.79	16.21	9.78
	Range	4–94	0–71	0–39
	Reduction (%)^e^		50.24	67.43
Spinosad^c^	# Dogs	26	26	25
	Mean CADESI - 4 Score^d^	32.65	16.58	12.28
	SD	20.50	13.43	9.60
	Range	1–91	1–58	3–43
	Reduction (%)^e^		49.23	62.39

*Abbreviation: SD* standard deviation

No significant differences between the two groups were observed, for any day, based on the repeated measures analysis (*P* = 0.8588)

^a^In both groups, dogs were treated on day 0 and once between days 56–60

^b^Dogs were orally administered Sarolaner chewables (Simparica® Zoetis) according to label directions

^c^Dogs were orally administered Spinosad flavored tablets (Comfortis® Elanco) according to label directions

^d^Arithmetic mean Canine Atopic Dermatitis Extent and Severity Index (CADESI)-4 scores [34]

^e^{(Day 0 arithmetic mean CADESI-4 score – day x arithmetic mean CADESI-4 score) / day 0 arithmetic mean CADESI-4 score)} × 100

Pruritus severity as assessed by owners improved rapidly in both treatment groups. On day 0, dogs administered sarolaner or spinosad had mean pruritus (PVAS) scores of 7.3 (range 1.3–10.0) and 7.4 (range 3.7– 10.0), respectively (Table 5). By day 7 post-treatment mean pruritus scores had dropped by 62.2% (2.76) and 56.5% (3.23) in the sarolaner and spinosad treatment groups, respectively (Table 5). At the final assessment conducted between days 56 to 60 the mean pruritus scores in both groups had dropped to 0.8 (range 0–4.1) and 1.1, (range 0–7.3) in the sarolaner and spinosad treatment groups, respectively (Table 5). Sarolaner and spinosad produced statistically significant reductions in PVAS scores across time (*P* < 0.0001), but no statistically significant difference between treatment groups (*P* = 0.6942) was observed. It is of interest to note that only 28 of 29 dogs in the sarolaner treatment group and 25 of 26 dogs in the spinosad treatment group had their PVAS scores included in the analysis. Pruritus scores from those two dogs were excluded when it was discovered at the end of the study that more than one person in those households had been providing pruritus assessments during the study.

**Table 5:** Owner assessment of pruritus using a visual analogue scale (PVAS) for dogs naturally infested with fleas and administered either Sarolaner or Spinosad treatments

Treatment group		Days post-treatment^a^					
		Day 0	Day 7	Day 14	Day 28–30	Day 42–45	Day 56–60
Sarolaner^b^	# Dogs	28	28	28	28	28	27
	Mean PVAS Score^d^	7.30	2.76	2.43	2.25	1.25	0.83
	SD	2.22	2.67	2.39	2.23	1.73	1.15
	Range	1.3–10.0	0–9.8	0.2–9.5	0–6.5	0–7.8	0–4.1
	Reduction (%)^e^		62.23	66.68	69.18	82.88	88.64
	# (%) ≤ 1.9	2 (7.14%)	13 (46.43%)	16 (57.14%)	13 (46.43%)	21 (75.0%)	23 (85.19%)
Spinosad^c^	# Dogs	25	25	24	25	25	23
	Mean PVAS Score^d^	7.43	3.23	2.69	2.85	1.58	1.07
	SD	1.61	2.21	2.50	2.40	1.79	1.75
	Range	3.7–10.0	0.1–7.6	0–9.5	0–8.8	0–7.8	0–7.3
	Reduction (%)^e^		56.54	63.76	61.66	78.73	85.60
	# (%) ≤ 1.9	0 (0.0%)	8 (32.00%)	13 (54.17%)	11 (44.00%)	21 (84.0%)	20 (85.60%)

*Abbreviation: SD* standard deviation

No significant differences between the two groups were observed, for any day, based on the repeated measures analysis (*P* = 0.6942)

^a^In both groups, dogs were treated on day 0 and once between days 56 – 60

^b^Dogs were orally administered Sarolaner chewables (Simparica® Zoetis) according to label directions

^c^Dogs were orally administered Spinosad flavored tablets (Comfortis® Elanco) according to label directions

^d^Arithmetic mean pruritus score as assessed by dog owners using the PVAS [31]

^e^{(Day 0 arithmetic mean PVASscore – day x arithmetic mean PVAS score)/day 0 arithmetic mean PVAS score)} × 100

When homes within treatment groups were averaged together there was a shift over time in the ratio of female to male fleas collected in premises flea traps. On day 0, prior to treatment, 52.9 and 57.1% of the fleas collected in intermittent-light flea traps, in the sarolaner and spinosad treatment groups respectively, were females. Then on days 7, 14, 21, 28–30 and 40–45 days in the sarolaner treatment group females collected in the intermittent light traps accounted for 37.8%, 43.1%, 28.3%, 27.5%, and 28.6% of the population, respectively. No fleas were collected in traps in these homes during the last collection period. In the spinosad treatment group the percent of female fleas collected in traps also decreased over time with 48.5%, 39.5%, 43.2%, 41.7%, 0% and 0% of fleas being females on days 7, 14, 21, 28-30, 40–45 and 56–60, respectively.

Client interviews clearly showed that reservoir hosts for *C. felis* were commonly observed by pet owners. Many pet owners said they had seen opossums (45.7%; 16/35), raccoons (45.7%; 16/35) and/or feral cats (91.4%; 32/35) in their yards.

There were a few adverse events reported in dogs during the study period. In the sarolaner treatment group, one owner reported that a dog vomited within 1 h of product administration on day 0, dog was redosed with no subsequent vomiting. In the spinosad treatment group there were several adverse events reported. One owner reported that their dog had “loose stools” for 2–3 days following treatment on day 0 and three other owners reported their dog vomited within 1 h to “several” hours following administration of spinosad. The one dog that vomited within 1 h was redosed with no subsequent vomiting. These five (one for sarolaner and four for spinosad) events were determined to be likely caused by administration of the products.

Another owner reported that their dog was coughing several days after administration of the second dose of spinosad. This dog had a documented history of intermittent cough prior to being placed on study and was treated with an antitussive and signs resolved. Another owner reported that their dog died after eating some type of “red berries” nine days post-administration of spinosad on study day 0. Owner reported the dog had “bloody” diarrhea prior to death. The body of the dog was collected, necropsy performed and tissue samples submitted to an independent contract laboratory for analysis. Exact cause of death could not be determined, but the pathology report stated intestinal lesions were possibly from an enteropathogenic bacterial infection. Neither of these adverse events were deemed to have been likely caused by test drug.

## Discussion

Following the administration of two monthly doses of sarolaner chewables to dogs, a 99.0% reduction in geometric mean area flea counts was achieved within one week and almost total elimination (99.9%) of on-animal flea burdens occurred within 8 weeks. There was only one flea observed on one dog at the 8-week evaluation. The spinosad chewables also markedly reduced flea populations. The administration of two monthly oral doses of spinosad, reduced geometric mean area flea counts on dogs by 99.8% by days 56 to 60.

The area count methodology employed in this and previous in-home investigations conducted by these authors, has been documented to detect approximately 23.5% of the total pet flea burden [30]. Therefore, total body flea burdens of dogs prior to the administration of either sarolaner or spinosad based on geometric means area counts of 24.1 and 25.7, can be estimated to be 102.6 (range 21–506) and 109.4 (range 30–545), respectively. These numbers are clearly indicative of substantial natural flea infestations.

Another important evaluation criterion in these in-home investigations is the percentage of dogs at various time points that are completely free of fleas. While flea challenges year to year are clearly different, comparisons across years of studies conducted in west-central Florida can provide some perspective. In a 2010 study where dogs were administered either a dinotefuran-pyriproxyfen or fipronil (s)-methoprene topical spot-on formulation twice at monthly intervals, only 60.0 and 55.6% of dogs had no observable fleas by the end of the 2-month study [20]. In 2013 after two monthly applications of topical indoxacarb or fipronil (s)-methoprene 77.1 and 15.6% of the dogs had no observable fleas by the end of that 2-month study, respectively [22]. Then in a 2015 study when dogs were treated with either fluralaner or afoxolaner oral chewables 100 and 96.2% of the dogs were free of fleas at 8 weeks, respectively [27]. During the current study there was only one flea found on one dog (27/28; 96.4%) in the sarolaner treatment group at the end of this 8-week study. In the spinosad treatment group 92% (23/25) of the dogs were observed to be flea free at the end of the study. The very high efficacy combined with the percent of dogs with no observable fleas indicate that these orally administered systemically active products produced a rapid residual kill of fleas under natural exposure conditions.

A different measure of product performance entails the evaluation of gender structure of newly emerged (unfed) fleas collected in intermittent-light traps in these homes [19, 23]. While most insect species exhibit protrandry (males tend to emerge before females), *C. felis* exhibits protogny (females tend to develop before males) [19, 23]. From a given cohort of eggs female fleas are the first to emerge, followed by both females and males and then lastly mostly males. It has been previously documented that if flea reproduction is inhibited by insecticidal and/or insect growth regulator treatments administered to a pet, then a gender shift in premises flea population takes place overtime from a female dominated population towards a more male dominated population [19, 23]. In this study, only 47.1 and 42.9% of the fleas collected on day 0 in the two treatment groups were male, whereas by 40–45 days following treatment 71.4 and 100% of the fleas collected in the sarolaner and spinosad treatment group homes, respectively were male. This gender shift is indicative of a dramatic if not complete cessation, of flea reproduction once the products were administered.

In the current study, we also attempted to assess the effect of these orally administered flea adulticides on reducing dermatologic lesions and pruritus. The owner reported pruritus assessment tool (PVAS) we used has been previously validated and employed in field trials [21, 27, 31, 32]. Determination of the level of pruritus in a dog is subjective, but clinically important when attempting to determine the effectiveness of prescribed treatments in a patient with pruritic dermatitis [31, 32]. It has been previously suggested that in this pruritus scoring system a “normal” dog has a PVAS at or below 1.9 [32]. Using that criteria only 2 of 53 (3.8%) dogs for which we gathered reliable data had normal pruritus prior to treatment. Because the minimal on-animal flea burden is 5 fleas in total area counts, a dog likely has a total body flea burden of at least 21 fleas to qualify for entry into the study. In a 2015 in-home flea product investigation conducted in west central Florida using identical criteria all 61 dogs had PVAS > 1.9. It appears from these two studies that dogs with > 21 fleas are very likely (112/114; 98.2%) pruritic.

In this current study rapid improvement in PVAS was reported for patients receiving sarolaner or spinosad despite no additional therapies being allowed to manage pruritus. Within seven days of administration of sarolaner or spinosad chewables, 46.4 and 32.0% of dogs already had PVAS < 1.9, respectively (Table 5). Then at 8 weeks after two doses of either sarolaner or spinosad chewables, 88.2 and 85.6% of dogs had PVAS < 1.9, respectively (Table 5).

To evaluate skin lesions possibly associated with flea allergy dermatitis we used a flea bite hypersensitivity lesional scoring system employed in a previously conducted FAD induction study and used in a recently published in-home flea product investigation [27, 33]. The International Committee on Allergic Diseases of Animals (ICADA) currently recommends using either the CADESI-4, or, canine atopic dermatitis lesion index (CADLI) severity scales to assess and score dermatologic lesions in canine atopic dermatitis patients [34]. These authors chose to use CADESI-4 in this study because it provides a more thorough assessment of dermatologic lesions. It is important to remember that when dogs were being enrolled into this study no attempt was made to make a definitive diagnosis of FAD or CAD. The study was designed so that a single treatment-blinded investigator could utilize the two scoring systems to determine the clinical relevance of flea control on skin lesions when no additional treatments were allowed.

A clinical diagnosis of flea allergy dermatitis is frequently based on history, clinical signs, and response to anti-flea therapy [35]. Accuracy, specificity and sensitivity of whole-body flea extracts used for intradermal testing varies, particularly if conducted in flea endemic regions [35]. In this 2016 in-home investigation, flea bite hypersensitivity severity scores improved markedly in both treatment groups. By the end of the 8-week study there was a 66.2 and 62.1% improvement with sarolaner and spinosad, respectively. The pronounced decrease in PVAS as well as the FAD scores following treatment with sarolaner or spinosad indicates that most dogs enrolled in this study had clinical signs attributable to flea exposure.

Diagnosis of canine atopic dermatitis (CAD) is primarily a diagnosis of exclusion and is based on clinical signs, history and excluding other pruritic diseases that share similar characteristics [36, 37]. In this current study many of the enrolled dogs had skin lesions consistent with those observed in dogs with atopic dermatitis, however further evaluation would have been necessary for this diagnosis to be made. Following administration of the oral flea adulticides, improvement in CADESI-4 scores was clearly evident. The marked reduction in CADESI-4 scores, might lend support to the hypothesis of an allergic threshold for the development of atopic dermatitis. When allergen levels are low, symptoms may not be observed, however as allergen levels rise above a specific threshold, clinical disease results [36]. In the current study, rapid and almost complete elimination of fleas resulted in marked improvement in PVAS, CADESI-4, and FAD scores in these dogs, without the benefit of additional treatments.

## Conclusions

This in-home flea product evaluation conducted during the summer of 2016 in subtropical west central Florida showed that the monthly administration of an oral chewable containing sarolaner effectively controlled flea populations on dogs and in the indoor premises. Spinosad, as a positive control, showed similar results. Not only were these flea infestations rapidly controlled by these oral systemic flea adulticides, but significant reductions in dermatologic lesion and pruritus scores were also observed without the use of any concurrent medical intervention.

## Abbreviations

CAD: Canine atopic dermatitis
CADESI: Canine atopic dermatitis extent and severity index scoring system
CADLI: Canine atopic dermatitis lesion index
FAD: Flea allergy dermatitis
ICADA: International Committee on Allergic Diseases of Animals
LSMeans: Least Square Means
PVAS: Pruritus visual analogue scale
